# Descriptive Sensory Profile and Consumer Study Impact of Different Nutritive and Non-Nutritive Sweeteners on the Descriptive, Temporal Profile, and Consumer Acceptance in a Peach Juice Matrix

**DOI:** 10.3390/foods11020244

**Published:** 2022-01-17

**Authors:** Alessandra Medeiros, Elson Tavares, Helena Maria Andre Bolini

**Affiliations:** Department of Food Engineering and Technology, University of Campinas, Monteiro Lobato, 80, Campinas 13083-862, SP, Brazil; acmls@unicamp.br (A.M.); elsontavares@live.com (E.T.)

**Keywords:** descriptive quantitative analysis, time–intensity analysis, stevia, natural sweetener, peach juice, PLS regression

## Abstract

The study evaluated the effect of peach juice sweetened with sucrose, widely used non-nutritive sweeteners, the artificial sucralose, neotame blend, and the natural stevia extract with different rebaudioside A concentrations on the temporal and quantitative descriptive profile, and consumer acceptance of the beverage. The sensory profiling was determined by quantitative descriptive and time–intensity analyses. The results showed that the sweeteners neotame and sucralose present higher sweetening power, and the different rebaudioside A concentrations did not affect the sweetening power of the stevia extract. The samples sweetened with stevia with 40% and 95% of rebaudioside A were characterized by the sensory attributes bitter taste, bitter aftertaste, astringency, and black tea flavor, with a negative influence on the consumers’ acceptance. The different concentrations of rebaudioside A in stevia interfered substantially in the descriptors bitter taste and bitter aftertaste, showing that the higher the percentage of rebaudioside A, the lower bitterness of peach juice.

## 1. Introduction

Peach (*Prunus persica* L. Batsch) fruit is native to China and widely cultivated in the temperate climate zone in eastern Europe, and the Americas. Peach is the eighth-most produced fruit in the world [1], with seasonal production and great importance, being a source of carotenoids, such as β-carotene and β-cryptoxanthin, with protective effects due to its antioxidant properties [2].

Fruit preservation in the form of juice can increase the fruit supply and enable the use of surplus production [3]. Thus, the importance and popularization of ready-to-drink juices have gained prominence due to the ease of consumption, availability of fruit outside the local harvest season, in addition to the nutritional quality and health benefits of the beverage [4]. However, juices, like other processed foods, contain added sugar, which has contributed to the increased incidence of obesity. It is known that obesity is related to excessive consumption of calories from sugars, such as glucose, fructose, and sucrose, among other factors. Within this context, sucrose replacement by sweeteners represents an alternative for consumers to reduce their energy intake [5].

The search for alternatives to replace sucrose has encouraged studies on the production of attractive formulations for consumers of light and diet products [6]. Several sweeteners have been approved for use by JECFA (Joint FAO/WHO Expert Committee on Food Additives) [7], most notably sucralose, acesulfame potassium, aspartame, cyclamate, saccharin, thaumatin, neotame, and steviol glycoside. Steviol glycoside appears as an alternative to synthetic sweeteners. It is a sweetener of natural origin extracted from the leaves of *Stevia rebaudiana* (Bert.), a branched shrub belonging to the Asteraceae family, cultivated in several regions of the world [8,9].

Of the 230 species in the genus Stevia, only the species *rebaudiana* and *phlebophylla* produce steviol glycosides [10]. Stevioside is one of those responsible for the sweet taste coming from stevia, but it also has a pronounced bitter aftertaste, narrowing its use in large quantities [11,12,13]. Rebaudioside A has higher sweetening power when compared to stevioside, and is stable and less bitter [14].

Studies have focused on increasing the rebaudioside A concentration to improve the sensory characteristics and mask the bitter aftertaste, producing leaves containing rebaudioside A from 40% to 97% purity [15].

The sweetening power of steviol glycoside can vary according to the ratio of stevioside to rebaudioside in the *Stevia rebaudiana* extract and purity [16], as well as the duration of the sensory stimuli, such as bitterness, impacting its use by the food industry.

Several steviol glycosides were detected in stevia extracts from different suppliers, those being the more cited rebaudioside A, B, C, D, F, G, H, I, J, K, M, and N, stevioside, dulcoside A and B, ruboside, and steviolbioside [17]. The scientific literature cites that the more rebaudioside A the better [18].

Steviol glycosides are known for their intense sweetness. They are diterpene glycosides present in the *Stevia rebaudiana* Bertoni leaf. These compounds, mainly stevioside and rebaudioside, are known for their intense sweetness and are applied as non-caloric sweeteners in several countries.

Sweetening power, presence or absence of undesirable flavors, and sensory characteristics may vary depending on the sweetening agent in addition to the interaction of the food matrix where it is added to replace sucrose.

Researchers found similarities among the sweeteners’ profiles (aqueous solution) in a study to characterize the dynamic sensory properties of nutritive and non-nutritive sweeteners through a temporal check-all-that-apply [19]. Sixteen sweeteners in equisweet intensities to 10% *w*/*v* sucrose were studied using the same temporal method (TCATA) [20]. The authors found that sweeteners have distinct sensory, physical, nutrient, and metabolic characteristics that need observation when a sweetener is used to replace or reduce sucrose [21].

Sucrose, sucralose, erythritol, rebaudioside A, and tagatose were analyzed in relative concentrations of 3% to 20% sucrose [*w*/*v*] to determine the relative sweetness and potency of the sweeteners. The authors found that rebaudioside A was the only sweetener with high bitterness which became progressively intense with increasing concentration [22].

Published data have shown relevant results [23] that indicate that non-nutritive sweeteners are not supernormal stimuli. Moreover, the carbohydrate sweeteners present a receptor-mediated process and not linear functions. Published studies do not support the claim that non-nutritive sweetness produces harmful health effects by overstimulating sweet taste receptors to produce hyper-intense sweet sensations [21].

Growing health issues have increased the reduction in the consumption of added sucrose and other sugars, which can be possible by replacing the sucrose with sweeteners to preserve the intensity and sensory quality. The increase in studies and knowledge about sweeteners has increased, showing the importance relative to the intensity and temporal properties.

In foods, the sweetness could be altered by the taste–flavor interactions with the inherent tastings and possible matrix effects that can modify the release and perception of sweetness [18].

Within this context, sensory studies are necessary, especially those that concern the temporal profile of these compounds, aimed to develop products with greater consumer acceptability. Therefore, the objective of this study was to determine the quantitative and dynamic descriptive sensory profile by comparing natural sweeteners with those currently most used industrially to obtain a ready-to-drink beverage with high consumer acceptance.

## 2. Materials and Methods

### 2.1. Materials

Peach juice samples were prepared using frozen pasteurized peach pulp containing 12% natural sugar (DeMarchi^®^, Campinas, SP, Brazil) and mineral water in a 2:3 ratio. Five different sweeteners were used to replace sucrose (União^®^), as follows: sucralose (Sweetmix^®^, Campinas, SP, Brazil); neotame (Sweetmix^®^, Campinas, SP, Brazil); stevia extract with 40% and 95% rebaudioside A (Clariant^®^, São Paulo, SP, Brazil); and a blend containing acesulfame, sucralose, and neotame (100:50:1) (Sweetmix^®^, Campinas, SP, Brazil).

All sensory evaluation studies of peach juice were performed at the Laboratory of Sensory Science and Consumer Research (LSSCR) in the School of Food Engineering at the University of Campinas (UNICAMP).

Preliminary studies were performed to determine the ideal sucrose concentration (8.6%) in peach juice determined by just-about-right using the JAR scale. Then, the sweetness equivalent to the sweeteners in the same ideal sweetness was determined by the magnitude estimation method [24]. For this purpose, the peach juice was sweetened with sucrose at 5%, 7%, 9%, 11%, and 13%, *w*/*w* and analyzed by 120 consumers (61% female and 39% male with an age range of 22 to 38). The equisweet concentration relative to peach juice with 8.6% sucrose was carried out by 14 assessors (7 women and 7 men, 25–40 y old). The results of equisweet in the ideal sweetness of peach juice were: 0.0169% for sucralose, 0.0018% for neotame, 0.1055% for stevia extract 40% and 95% of Rebaudioside A, and 0.0332% for the acesulfame, sucralose, and neotame (100:50:1) blend [24].

The peach pulp, water, and sweetener quantity used in each sample is presented in Table 1.

### 2.2. Methods

#### 2.2.1. Participants

All participants of sensory analysis (preliminary tests, QDA^®^, TI, and consumers test) were recruited through social networks and email lists, from Sao Paulo and Campinas regions, Sao Paulo State, Brazil.

Participants signed an informed consent form before the evaluation. The study was approved by the Research Ethics Committee of the State University of Campinas (UNICAMP), Brazil (CAAE: 91178118.0.0000.5404).

#### 2.2.2. Quantitative Descriptive Analysis—QDA^®^

The pre-selected assessors defined 20 sensory descriptors for the peach juice samples using the Grid method [25] concerning the appearance, aroma, flavor, and texture, as well as maximum and minimum references (Table 2). All evaluated aroma descriptors were analyzed by the orthonasal method.

The sensory team was composed of 13 assessors (7 women and 6 men 25–40 y old), who were trained and selected based on the discrimination power between samples, repeatability, and agreement between the team [26] determined by two-factor analysis of variance (ANOVA) (sample and repetition) for each assessor and attribute. The assessors with Fsample (*p* < 0.50) and Frepetition (*p* > 0.05) values for each parameter were selected, and the agreement with the team and the non-significant interaction between sample and assessor (*p* > 0.05) was evaluated.

The samples were prepared immediately before the tests and maintained at refrigerator temperature for all test periods.

The six samples were presented to assessors in balanced block design in monadic presentation. Fifty milliliters of peach juice at a temperature of 10 °C ± 2 °C was offered in translucent plastic cups, coded with 3-digit numbers [27].

The tests were carried out in three repetitions, using a nine-centimeter unstructured scale, marked at the extreme on the left by the term “weak” or “none” = 0, and on the right by the term “strong” or “very” = 9 [27].

QDA^®^ data were collected using FIZZ Network Sensory software (version 2.47b) and analyzed by analysis of variance (ANOVA) and Tukey’s test at a 5% significance level using the SAS software (Statistical Analysis System, 2012—Version 9.4, Raleigh, United States of America) licensed to the University of Campinas.

#### 2.2.3. Time–Intensity Analysis

A time–intensity analysis (TI) was performed for the stimulus sweet taste and bitter taste. Thirteen assessors aged between 20 and 38 years were trained through direct contact with the minimum (zero) and maximum (9) intensity references of sweetness and bitterness to the formation of sensory memory. Deionized water was a reference to minimum intensity to both tastes. To maximum sweet taste, the reference was Peach juice and water (1:2) with sucrose (25%—União^®^) and to bitter taste was peach juice and water (1:2) with caffeine 0.20% Ecibra^®^.

In this step, the participants were also trained to use the data collection software Time Intensity Analysis of Tastes and Flavors (TIAFT).

##### Training and Selection of Assessors to Time–Intensity

The sixteen candidates for the time–intensity analysis were previously selected through the WALD sequential analysis [28] using triangular tests. The assessors were trained in at least three sessions of approximately one hour each, in the recognition of stimulus sweet and bitter, the formation of sensory memory of the maximum and minimum references for each attribute, use of the software, and records of the sensations perceived with precision and reliability.

After training, the candidates evaluated the six samples in a balanced complete block design in three repetitions. From sixteen pre-selected candidates, thirteen assessors were selected due to their discrimination power, repeatability, and agreement with the team [27]. The assessors with significant pF sample (*p* < 0.30), non-significant pF repetition (*p* > 0.05), and non-significant interaction between sample and assessor (*p* > 0.05) were selected for each parameter of curves (Imax, Timax, Ttot, and Area) in addition to a consensus with the team [27], sweetness and bitterness stimulus, validation of the training and obtaining an in the improvement in quality and validity of data acquisition.

##### Sweetness and Bitterness Time–Intensity Analysis

The thirteen selected assessors (basis on the method cited in Training and Selection of Assessors to Time–Intensity topic) participated in the definitive time–intensity analysis of the six samples of peach juice.

The samples were prepared immediately before the tests and maintained at refrigerator temperature.

For the analysis of sweetness and bitterness (separately), the six samples were presented to assessors in a balanced block design in a monadic way with three repetitions. Fifty milliliters of peach juice at a temperature of 10 °C ± 2 °C was offered in translucent plastic cups, coded with 3-digit numbers [29].

The data collection for the time–intensity analysis was carried out on a computer using a dynamic sensory profile using TIAFT, which was developed at the Laboratory of Sensory Science and Consumer Research of the School of Food Engineering [30]. The standardized conditions for analysis of the four attributes were as follows: (1) judge’s wait time, 10 s; (2) time with the sample in the mouth, 10 s; (3) time after swallowing, 50 s; and (4) intensity scales, 9.

The assessors evaluated each sample using a computer mouse to record the intensity of sensory characteristics on the scale according to the time. The software shows a continuous scale marked with 10 points with numbers (0 to 9) on the screen. The mouse cursor slides freely so that the trained assessor can continuously indicate the perceived intensity of the determined characteristics as a function of time. A horizontal continuous scale was used, with 10 vertical lines indicating the numbers 0 through 9. The scale was labeled such that 0 corresponds to none (far left), 4.5 corresponds to moderate (middle), and 9 corresponds to strong (far right). Data were continuously collected by the TIAFT software from the start until the conclusion of the test. A plot and table with each tenth of a second of analysis and its corresponding intensity were generated. The software time manager was pre-established for each specific food studied. In the present analysis, all conditions were standardized with the same time for the two attributes [28].

The parameters of curves Imax (maximum intensity), Timax (time to maximum intensity), time total of stimulus (Ttotal), and area under curve (Area) were analyzed [28] by analysis of variance (ANOVA) and Tukey’s test at a 5% significance level using the SAS software (Statistical Analysis System, 2012—Version 9.4, Raleigh, NC, USA) licensed to the University of Campinas.

#### 2.2.4. Consumer Test

One hundred twenty consumers (72 women and 48 men, aged 19–48) were recruited to participate in the acceptance test of peach juice with different sweeteners [31]. The selection criterion was that subjects had to consume peach juice at least once a week and be adults over 18 y of age.

The analysis was carried out in LSCRS, in an individual informatized booth. The consumers received the samples in a balanced block design in a monadic presentation. Fifty milliliters of peach juice at a temperature of 10 °C ± 2 °C was offered in translucent plastic cups, coded with 3-digit numbers [27].

Acceptance was determined concerning the overall liking using a 9-cm linear scale (not structured), with anchors of “dislike extremely” on the left and “like extremely” on the right. The subjects were instructed to rinse their mouths with distilled water between samples to avoid the carry-over effect. To prevent bias, no information about the samples was given to the consumers [17,29].

Correlation between the descriptor terms intensity obtained in QDA^®^ and consumer test data was applied by partial least square regression analysis (PLSR) [32] using the software XLStat 2015 (Addinsoft, Paris, France). The overall liking was the dependent variable (Y-matrix), and the QDA attributes were the independent variables (X-matrix).

## 3. Results and Discussion

### 3.1. Descriptive Quantitative Analysis

Table 3 presents the means of descriptor terms relative to each studied sample. The means were submitted to Tukey’s test. Means with the same letters on the same row did not differ at *p* ≤ 0.05 significantly.

Concerning the attribute appearance, significant differences were observed among the samples (*p* ≤ 0.05) for four descriptive terms as follows: yellow color, brightness, apparent viscosity, and presence of particles. For the attribute yellow color, only the sucralose-sweetened sample showed a significant difference in comparison to the sucrose-sweetened sample, with no difference among the other samples. For the attributes brightness and viscosity, only the sample sweetened with stevia with 40% of rebaudioside A showed higher and lower values, respectively, differing from the sucrose-sweetened sample. Concerning the presence of particles, the sucrose-sweetened sample showed higher scores, differing from the samples with stevia extract, for both rebaudioside A concentrations.

Regarding the orthonasal aroma descriptors, no significant differences were observed among the peach juice samples (*p* ≥ 0.05) for the descriptors peach aroma, sweet aroma, sour aroma, and cooked peach aroma.

Although the assessors recognized the orthonasal sweet aroma in reference to maximum intensity, they did not find differences of this same characteristic among the samples. It is probable the sucrose added to the peach juice (nectar) that has fructose as natural sugars [33] provides a sweet orthonasal aroma perception, but this difference is not perceived among the samples.

A substantial change was observed for the flavor descriptors when sucrose was replaced by sweeteners, with a significant difference (*p* ≤ 0.05) for all descriptors evaluated. The descriptive term peach flavor presented higher intensity for the sucrose-sweetened sample, not differing from the sample sweetened with the blend of sweeteners. The samples sweetened with stevia extract were similar (*p* ≤ 0.05) to the sucrose and sucralose sweetened samples concerning sweet taste, differing significantly (*p* ≤ 0.05) from the samples sweetened with neotame and blend of sweeteners, which exhibited higher sweetness intensity.

Regarding the sweet aftertaste, the neotame-sweetened sample differed from the other samples, showing higher intensity, while the sucrose and sucralose sweetened samples had lower averages, both differing significantly (*p* ≤ 0.05) from each other and the other samples. For the descriptors bitter taste and bitter aftertaste, the samples sweetened with stevia presented a significant difference (*p* ≤ 0.05) when compared to the other samples and between the different rebaudioside A concentrations. The sample sweetened with stevia at 40% rebaudioside A presented a higher intensity for both descriptors. Regarding the attribute sourness, only the sucralose-sweetened sample differed from the sucrose-sweetened sample, presenting a lower intensity.

Concerning the attribute cooked peach flavor, the sample sweetened with stevia at 40% rebaudioside A differed significantly (*p* ≤ 0.05) from the sucrose-sweetened sample, presenting a lower score, with no differences from the sample sweetened with 95% rebaudioside A. For the attribute astringency, the samples sweetened with stevia 40% and 95% rebaudioside A showed higher averages, differing from the samples sweetened with sucralose and a blend of sweeteners. Higher intensity of the attribute black tea flavor was observed for the sample sweetened with stevia with 40% rebaudioside A, not differing from the samples stevia rebaudioside A 95% and neotame. This result is due to the natural bitterness of black tea due to its high caffeine content and can be correlated with the bitterness coming from the stevioside.

The sucrose-sweetened sample showed higher scores for the attributes viscosity and cremosity, differing significantly (*p* ≤ 0.05) from the samples sweetened with stevia 40% and 95% rebaudioside A and neotame. For the attribute body, the sucrose-sweetened sample exhibited a significant difference when compared to the sample sweetened with sucralose and stevia with 95% rebaudioside A, with no significant differences among the other samples.

The peach juice sample with a blend of sweeteners showed sweetness, while the peach juice sample with neotame showed a sweeter aftertaste than the other samples (*p* ≤ 0.05). Stevia with 40% rebaudioside A was the sweetener that conferred the highest bitterness and bitter aftertaste (*p* ≤ 0.05), followed by Stevia with 97% rebaudioside A.

The sucrose-sweetened sample presented greater distinction, which was characterized by the descriptors peach flavor not differing from the blend-sweetened sample (*p* > 0.05).

In general, the results of quantitative descriptive analysis showed that the peach juice samples sweetened with sucralose and the blend of acesulfame-K/sucralose/neotame (100:50:1) had a sensory profile with more proximity to sucrose-sweetened sample. Although the different concentrations of rebaudioside A in the stevia extract did not interfere in the other descriptors, it substantially interfered in bitter taste and aftertaste, decreasing the intensity with increased rebaudioside A concentration (*p* ≤ 0.05).

Undesirable descriptors, such as bitter taste and aftertaste, can diminish the perception of some sensory descriptors, such as fruit flavor, mischaracterizing the product.

### 3.2. Time–Intensity Analysis

**Figure 1:**
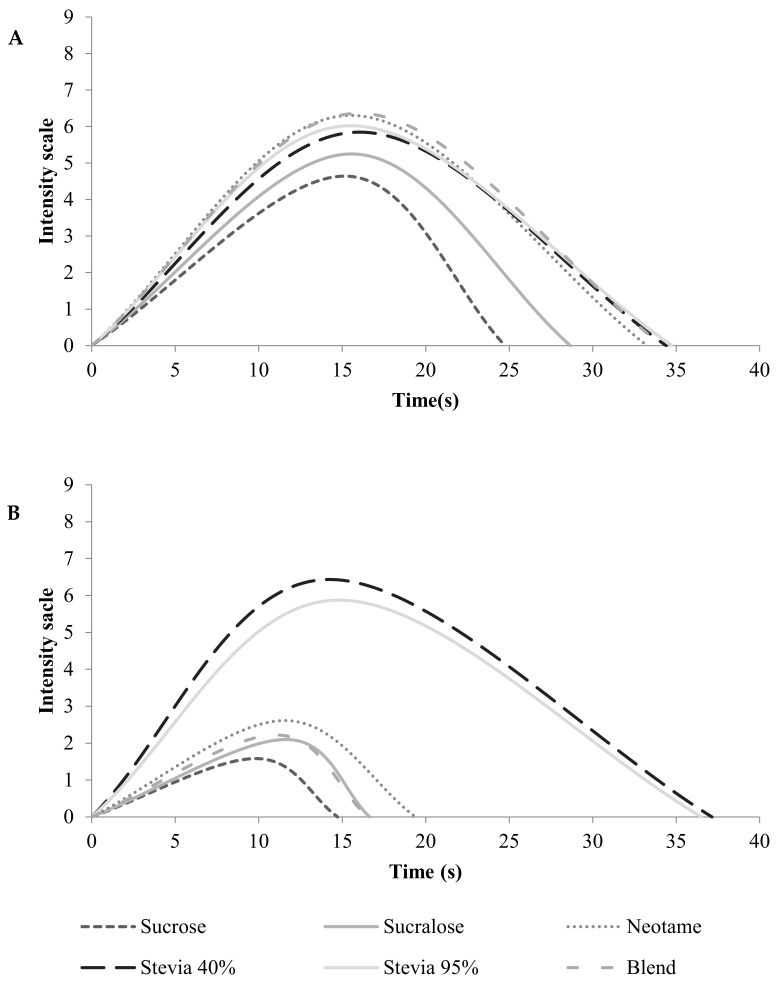
Time–intensity curves relative to the sweetness (**A**) and bitterness (**B**) for samples of peach nectar.

Table 4 presents the means of parameters curves for stimuli sweet (A) taste and bitter (B) taste. The averages were used to construct a time–intensity curve of peach juice samples relative to the sweet taste (stimulus) and bitter taste (stimulus), respectively, Figure 1A,B.

#### 3.2.1. Sweet Taste

The results obtained for the sweet stimulus indicated no significant differences (*p* ≥ 0.05) for the time to maximum intensity (Timax), thus with no differences in the time to the perceived maximum intensity of the stimulus. Concerning the parameter maximum intensity (Imax), no significant difference was observed for the different rebaudioside A concentrations in the stevia extract; however, the sample sweetened with stevia with 40% rebaudioside A differed significantly from the other samples (*p* ≤ 0.05). For the parameters area (Area) and total duration time (Ttot), the sucralose-sweetened sample differed significantly from the other samples sweetened with sweeteners, showing a profile closer to the sucrose-sweetened sample for the sweet taste stimulus (*p* ≤ 0.05).

The samples sweetened with neotame, blend of sweeteners, stevia with 40% and 95% rebaudioside A indicated the parameters area, total time, time to maximum intensity, and maximum intensity did not differ significantly (*p* > 0.05), except the Imax for Stevia Reb A 40% and Blend. The sample sweetened with stevia with 40% rebaudioside A was associated with time to maximum intensity.

The peach juice sweetened with sucrose presented maximum intensity and area of sweetness (Table 4A) curves lower than other samples. Concerning bitterness, peach juice sweetened with sucrose presented a maximum intensity score lower (Table 4B) than other samples (*p* ≤ 0.05).

In this study, the peach juice sucrose-sweetened did not differ from the other samples (Table 4A) concerning time to maximum intensity of sweetness (*p* > 0.05).

**Figure 2:**
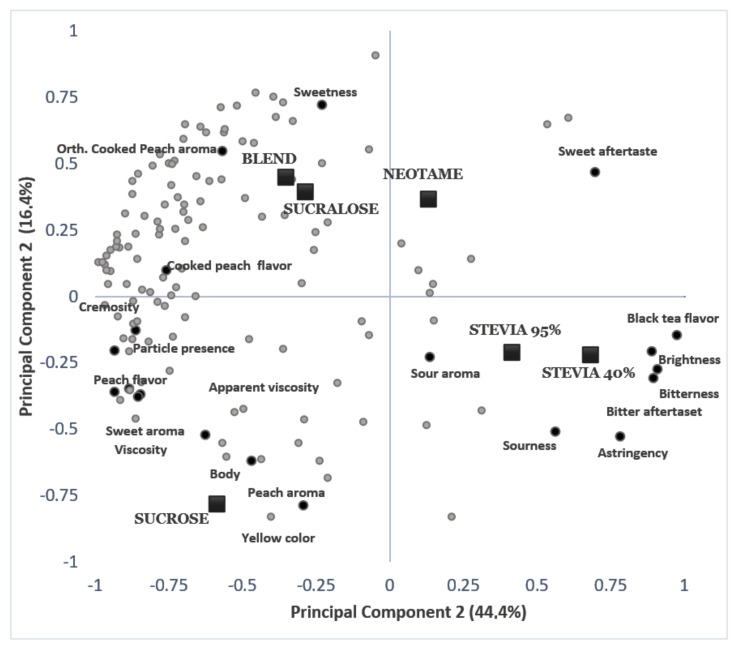
External preference mapping obtained by partial least squares regression of the descriptive sensory profile and consumers’ overall impressions of the functional peach juice. (Square = samples; grey points = consumers; black points = quantitative descriptive analysis attributes).

The joint analysis of ANOVA, Tukey’s tests (Table 4A,B), and time–intensity curves of each sweetener (Figure 2) indicate no significant differences between the samples sweetened with stevia 40% and 95% rebaudioside A in the temporal profile of the sweet taste stimulus.

The sucrose and sucralose sweetened samples had lower intensity and duration of the stimulus and were not distinguished by the presence of sweet aftertaste. The results of the time–intensity analysis for the sweet stimulus showed that the sucralose-sweetened sample did not differ from the sucrose-sweetened sample for total time of duration (*p* > 0.05). Similar results were found in mango nectar [34], pitanga nectar [35], and frozen [33] dessert chocolate in which sucralose presented the temporal sweetness similar to sucrose.

Although the stevia-sweetened samples showed a temporal profile for sweet taste different from that found for peach juice sweetened with sucrose, similarities were observed when compared to other sweeteners commonly used in the food industry, making it a viable alternative to non-nutritive sweeteners.

#### 3.2.2. Bitter Taste

Significant differences (*p* ≤ 0.05) were observed for the bitter taste between all samples in the time–intensity curve (Figure 2). The samples sweetened with stevia with 40% and 95% of rebaudioside A presented the highest scores for all parameters studied, with significant differences (*p* ≤ 0.05) to the other samples. However, for the parameters maximum intensity and total area, the stevia-sweetened samples showed a significant difference among them, with higher scores observed for the sample sweetened with stevia with 40% rebaudioside A, which also exhibited higher scores for the bitter taste and bitter aftertaste in the quantitative descriptive analysis (Table 3).

Significant differences (*p* ≤ 0.05) were observed for the parameters maximum intensity and total area among the other samples, with lower scores for the sucrose and sucralose sweetened samples, indicating that these sweeteners provided low bitterness. Regarding the total area, the neotame sweetened sample stood out, differing significantly from the other samples. The pitanga nectar sweetened with sucrose and sucralose showed similar results, with lower averages for this parameter [35].

Regarding bitterness time–intensity profile, the sample sweetened with Stevia Reb A 40% showed maximum intensity and area higher than sample sweetened with Stevia Reb A 95%, evidencing a likely attenuation of these parameters in the bitterness time–intensity profile with an increase in the percentage of rebaudioside A in this sweetener (*p* ≤ 0.05), in concordance with scientific literature [36]. Stevia Reb A 40%, Stevia Reb A 97%, neotame, and blend used as sweeteners in peach juice presented total time and area (*p* ≤ 0.05).

The peach juice sweetened with stevia with 40% rebaudioside A differed from the other samples for higher maximum intensity and area (*p* ≤ 0.05) of the bitterness curve. These results were in concordance with the descriptive sensory profile that showed the descriptors bitterness and bitter aftertaste being higher in the sample sweetened with Stevia Reb A 40% than the other sweeteners Table 2.

The joint analysis of ANOVA, Tukey’s tests (Table 2), and time–intensity curves of each sweetener (Figure 1B) indicated that the samples sweetened with stevia at 40% and 95% rebaudioside A stood out regarding the intensity and duration of the bitter stimulus, characterized by a bitter taste and bitter aftertaste. However, the sample sweetened with stevia with 40% rebaudioside A presented a higher duration and intensity of the stimulus, differing significantly (*p* ≤ 0.05) from the sample sweetened with stevia with 95% rebaudioside A, indicating that the higher the rebaudioside A concentration, the lower bitterness of peach nectar. The sucrose-sweetened sample showed lower values for duration and intensity of the stimulus and was also characterized by a low bitter aftertaste, possibly coming from the fruit itself.

### 3.3. Consumer Analysis

The average scores given by consumers for the acceptance concerning appearance, aroma, flavor, texture, and overall liking are shown in Table 5.

This study allowed the identification of descriptors with significant differences (*p* ≤ 0.05) between samples and consumer acceptance. For the attribute appearance, no significant difference was observed (*p* ≤ 0.05) among the samples. Regarding the acceptance in relation to aroma, flavor, and texture, the samples sweetened with stevia at 40% and 95% rebaudioside A showed lower scores, with no significant differences between them, but differing from the other samples (*p* ≤ 0.05). For the overall liking, the samples sweetened with sucrose, sucralose, and a blend of sweeteners showed higher consumer acceptance, not differing significantly (*p* ≥ 0.05) from each other. No significant differences were observed for the samples sweetened with stevia with 40% and 95% rebaudioside A, which exhibited lower scores, with values below 4.5, indicating consumer rejection. Probably this fact can be explained by the bitterness and bitter aftertaste being significantly higher (*p* ≤ 0.05) in the samples with these sweeteners. It is very interesting to highlight that the time–intensity profile of both sweeteners is more intense in maximum intensity and total time than the other sweeteners, a fact that is evidenced only by the application of time–intensity analysis.

Although the time–intensity profile showed that the intensity and duration of bitterness were higher in the sample sweetened with 40% rebaudioside A stevia than 97%, both samples did not differ significantly in overall liking, showing that for consumers, the bitterness at either intensity renders rejection.

### 3.4. ADQ and Acceptance Data Correlation

**Figure 3:**
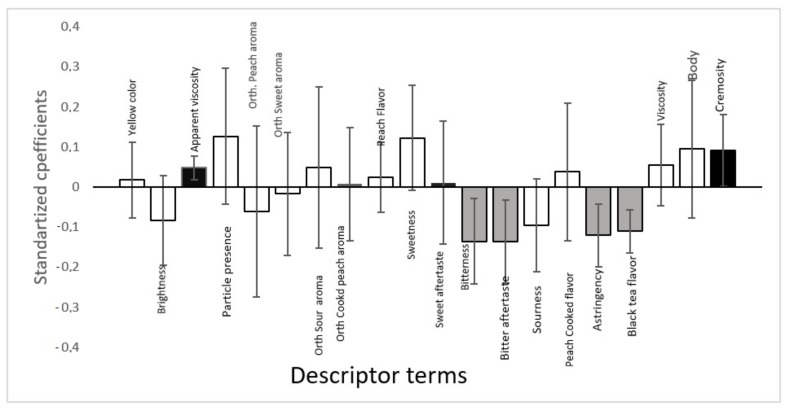
Partial least squares standardized coefficients of peach juice with sweeteners (black, descriptor terms that contribute positively to consumer acceptance; white, descriptive terms that did not significantly contribute to consumer acceptance; grey, descriptor terms that contribute negatively to consumer acceptance) 95% confidence interval.

The results of the overall liking were correlated with the ADQ^®^ descriptors terms intensity through multivariate analysis partial least square regression [32]. The standardized coefficients were obtained by partial least squares regression analysis (PLSR) to evaluate the impact of the sensory attributes on consumer acceptance. The confidence interval was 95%, and the PLSR results are shown in Figure 2 (to each one consumer data to overall liking) and Figure 3 (for consumer’s means to overall liking).

The external preference map (Figure 2), obtained through the principal components 1 and 2, explained 60.8% of the variation among the samples for acceptance. It was possible to observe the proximity of consumers with the samples sweetened with sucrose, sucralose, and a blend of sweeteners, indicating a preference for these samples.

The external preference map (Figure 2) showed the proximity of consumers with the samples sweetened with sucrose, sucralose, and a blend of sweeteners, indicating a preference for them.

The sucrose-sweetened sample was characterized by the descriptors yellow color, peach flavor, orthonasal sweet aroma, apparent viscosity, viscosity, peach flavor, and mouthfeel. The samples sweetened with sucralose and a blend of sweeteners were characterized by the descriptors sweet taste and cooked peach flavor. Neotame was positioned in an intermediate position to consumer preference, characterized by the sweet aftertaste, which may have influenced the consumers’ acceptance.

The samples sweetened with stevia 40% and 95% rebaudioside A were close to each other and distanced from the majority of the assessors, indicating consumer rejection for these samples, which presented lower scores for overall liking. The stevia-sweetened samples were characterized for the descriptors sour taste, sourness, astringency, black tea flavor, bitter taste, and aftertaste.

These results were consistent with the results found in the external preference map (Figure 2) in which the samples were characterized by the same descriptors. Analogous results were observed in acerola nectar [37], in grape nectar [38], and in passion fruit juice [39], who reported that consumers showed good acceptance for the sucrose and sucralose sweetened samples, and rejection of the stevia-sweetened samples for the descriptors flavor and overall liking.

The PLS regression analysis (Figure 3) allowed determining the descriptors that positively and negatively influenced the consumers’ acceptance. The columns represent the sensory attributes, and the extent of the columns represents the importance of each attribute to the consumer, whether negative or positive. The descriptors with a confidence interval below zero (negative region of the *Y*-axis) showed a negative effect for the score assigned to the overall liking, while the descriptors with the confidence interval above zero (positive region of the *Y*-axis) showed a positive effect [32].

In contrast, the descriptors that positively affected the acceptance of peach nectar were apparent viscosity and cremosity, which were assigned to the sucrose sweetened sample, according to the external preference map and quantitative descriptive analysis. Although the other positive descriptors were desirable in peach nectar, they did not affect the consumers’ acceptance.

A negative effect was observed for the descriptors bitter taste, bitter aftertaste, astringency, and black tea flavor. These terms were responsible for the characterization of the samples sweetened with stevia at 40% rebaudioside A and stevia at 95% rebaudioside A, according to the external preference map. This result explains the rejection of these samples by the consumers. The other negative descriptors are known as undesirable descriptors in peach nectar but did not affect the consumers’ acceptance.

The comparison of these results with those published with juices from other fruits [34,35,37,38,39] or other food matrices [36] can help to evidence that the same sweetener can confer different sensory characteristics in different beverages. These issues may influence the fruit juice’s acceptance and should be considered when substituting sucrose with sweeteners. It is important to highlight that the ideal sweetness for consumers found in mango juice was 7.0% [34], in pitanga and acerola, it was 8.0% [35,37], in passion fruit 9.4% [39], and grape juice 6.7% for smokers and 5.6% for non-smokers [38].

Although studies have been carried-out concerning sucrose replacement for ideal sweetness by sweeteners in the same sweetness-equivalent in mango, pitanga, acerola, and passion fruit juices, the present study specific to peach juice is an important result, because the fruits present high differences in chemical composition, such as ascorbic acid, total natural sugars, and total solids contents, as well sourness and pH [33].

## 4. Conclusions

The results showed that the different rebaudioside A concentrations in stevia extracts substantially interfered in the sensory profile of peach nectar, with an impact on the descriptors bitter taste and bitter aftertaste, which decreased with an increase in the concentration of rebaudioside A.

The results of the time–intensity analysis for the sweet stimulus of peach juice showed that the sucralose-sweetened sample presented a sensory profile closer to the sucrose-sweetened sample but was not characterized by the sweetness intensity and duration of the stimulus. The time–intensity analysis for the attribute bitter taste showed that the samples sweetened with sucralose and blend of acesulfame-K, sucralose, and neotame (100:50:1) presented a sensory profile closer to the sucrose-sweetened sample and were characterized by a low intensity of bitter taste and bitter aftertaste. The sample sweetened with stevia at 40% rebaudioside A showed higher bitterness intensity and duration, with differences from the sample sweetened with stevia at 95% rebaudioside A, showing that the higher the rebaudioside A concentration, the lower bitterness intensity in peach nectar.

The samples sweetened with sucrose, sucralose, and a blend of acesulfame-K, sucralose, and neotame (100:50:1) were better accepted by consumers in the acceptance test.

The present results show the importance of studying sweeteners in different products and formulations, emphasizing the important role of sensory evaluation in the development of novel food formulations.

## Figures and Tables

**Table 1:** Peach pulp *, water, and sweetener quantities to the six samples analyzed.

Sweeteners Added to Peach Juice	Peach Pulp (g)/100 mL	Water (mL)	Sweetener (*w*/*v*) g/100 mL
Sucrose	40.00	60.00	8.60
Sucralose	40.00	60.00	0.0169
Neotame	40.00	60.00	0.0018
Stevia Rebaudioside A 40%	40.00	60.00	0.1055
Stevia Rebaudioside A 95%	40.00	60.00	0.1055
Acesulfame, sucralose, and neotame (100:50:1) blend	40.00	60.00	0.0332

* Peach pulp has 12.00% of natural sugar (fructose).

**Table 2:** Descriptors terms, definitions, and references for peach juice samples.

Descriptor Terms	Definitions	References
Yellow color	Yellow color characteristic of products made with peach	Weak: Concentrate peach juice Maguary^®^ and water (1:2) Strong: Pure concentrate peach juice Maguary^®^
Brightness	Light reflection capability	Weak: boiled egg yolk Strong: Gelatina peach flavor Dr. Oetker^®^
Apparent Viscosity	Flow velocity on the wall glass cup	Weak: Concentrate peach juice Maguary^®^ and water (1:2) Strong: Peach pulp DeMarchi^®^
Particle Presence	Presence of particles/residues after draining the glass cup	Weak: Concentrate peach juice Maguary^®^ and water (1:2) Strong: Peach pulp DeMarchi^®^
Orthonasal Peach Aroma	Characteristic orthonasal aroma of fresh peach natural fruit	Weak: Concentrate peach juice Maguary^®^ and water (1:2) Strong: Peach pulp DeMarchi^®^
Orthonasal Sweet Aroma	Orthonasal sweet aroma from aromatic compounds that provide a sweet sensation	Weak: Concentrate peach juice Maguary^®^ and water (1:10) with sucrose (4%—União^®^) Strong: Juice peach (1:10) with sucrose (50%—União^®^)
Orthonasal Sour Aroma	Orthonasal sour aroma characteristic of the fermentation of peach juice	None: Concentrate peach juice Maguary^®^ and water (1:10) Strong: Pure concentrate peach juice Maguary^®^ at 22 °C by 4 days
Orthonasal Cooked peach aroma	Orthonasal peach aroma after the peach natural fruit by thermal processing	None: Concentrate peach juice Maguary^®^ and water (1:10) Strong: Canned peach in syrup Schramm^®^
Peach flavor	Orthonasal peach ripe flavor	None: Concentrate peach juice Maguary^®^ and water (1:10) Strong: Peach pulp DeMarchi^®^
Sweetness	Sweet taste characteristic of sucrose or sweetener	Weak: Concentrate peach juice Maguary^®^ and water (1:2) with sucrose (4%—União^®^) Strong: Concentrate peach juice Maguary^®^ and water (1:2) with sucrose (25%—União^®^)
Sweet aftertaste	Sweet taste that remains in the mouth after swallowing	Weak: Concentrate peach juice Maguary^®^ and water (1:2) Strong: Concentrate peach juice Maguary^®^ and water (1:2) sweetened with neotame 0.010% Sweetmix ^®^
Bitterness	Characteristic bitter taste of caffeine	Weak: Concentrate peach juice Maguary^®^ and water (1:2) Strong: Concentrate peach juice Maguary^®^ and water with caffeine 0.10% Ecibra^®^
Bitter Aftertaste	Bitter taste that remains in the mouth after swallowing	Weak: Peach juice and water (1:2) Strong: Peach juice and water (1:2) with caffeine 0.20%, Stevia 40% Rebaudioside A Sweetmix^®^
Sourness	Sour taste characteristic of unripe peach	Weak: Concentrate peach juice Maguary^®^ and water (1:2) Strong: Concentrate peach juice Maguary^®^ and water (1:2) with citric acid 0.10%
Cooked peach flavor	Peach flavor after thermal processing	Weak: Concentrate peach juice Maguary^®^ and water (1:2) Strong: Canned peach Schramm^®^
Astringency	Sensation of tannins in the mouth (such as green cashew)	Weak: Concentrate peach juice Maguary^®^ and water (1:10) Strong: Strong: peach pulp DeMarchi^®^
Black tea flavor	Characteristic flavor of black tea	None: Concentrate peach juice Maguary^®^ and water (1:10) Strong: Concentrate peach juice Maguary^®^ and water (1:2) with 25% black tea Nestea^®^
Viscosity	Perceived flow during swallowing of substances, such as Bordo syrup	None: deionized water Strong: Concentrate peach juice Maguary^®^
Body	Mouth-filling capacity, consistency of a drink	Weak: Peach juice and water (1:10) Strong: Peach pulp DeMarchi^®^
Cremosity	Flow of peach juice with a high proportion of pulp	Weak: Peach nectar and water (1:10) Strong: Vigor^®^ Greek yogurt yellow fruits flavor

**Table 3:** Attributes’ means * of descriptive sensory analysis for each peach juice sample (*n* = 39).

Attributes	Sucrose	Sucralose	Neotame	Stevia 40%	Stevia 95%	Blend	HSD **
Yellow color	5.63 ^a^	5.29 ^b^	5.45 ^ab^	5.38 ^ab^	5.40 ^ab^	5.33 ^ab^	0.31
Brightness	5.87 ^b^	5.86 ^b^	5.94 ^ab^	6.22 ^a^	6.01 ^ab^	5.90 ^b^	0.32
Apparent viscosity	4.58 ^a^	4.24 ^ab^	4.06 ^ab^	3.93 ^b^	4.06 ^ab^	4.29 ^ab^	0.6
Particle presence	3.93 ^a^	3.79 ^ab^	3.50 ^abc^	3.44 ^bc^	3.20 ^c^	3.64 ^abc^	0.44
Orthonasal peach aroma	5.52 ^a^	5.32 ^a^	5.23 ^a^	5.12 ^a^	5.42 ^a^	5.18 ^a^	0.52
Orthonasal sweet aroma	4.23 ^a^	4.06 ^a^	3.93 ^a^	3.90 ^a^	4.05 ^a^	4.13 ^a^	0.54
Orthonasal sour aroma	2.52 ^a^	2.28 ^a^	2.55 ^a^	2.54 ^a^	2.41 ^a^	2.53 ^a^	0.42
Orthonasal cooked peach aroma	4.24 ^a^	4.35 ^a^	4.21 ^a^	4.18 ^a^	4.27 ^a^	4.44 ^a^	0.41
Peach flavor	5.38 ^a^	4.76 ^bc^	4.62 ^c^	4.43 ^c^	4.71 ^bc^	5.11 ^ab^	0.46
Sweetness	4.16 ^b^	4.32 ^b^	5.17 ^a^	4.16 ^b^	4.18 ^b^	5.24 ^a^	0.53
Sweet aftertaste	0.66 ^d^	1.53 ^c^	4.48 ^a^	3.14 ^b^	3.17 ^b^	2.56 ^b^	0.66
Bitterness	0.33 ^c^	0.42 ^c^	0.52 ^c^	4.04 ^a^	2.76 ^b^	0.33 ^c^	0.59
Bitter aftertaste	0.22 ^c^	0.52 ^c^	0.60 ^c^	4.22 ^a^	2.97 ^b^	0.31 ^c^	0.6
Sourness	2.05 ^ab^	1.71 ^b^	2.08 ^ab^	2.11 ^a^	2.18 ^a^	1.95 ^ab^	0.39
Cooked peach flavor	4.30 ^a^	4.12 ^ab^	4.22 ^a^	3.77 ^b^	4.19 ^ab^	4.32 ^a^	0.43
Astringency	2.09 ^ab^	1.73 ^b^	2.12 ^ab^	2.41 ^a^	2.37 ^a^	1.91 ^b^	0.4
Black tea flavor	2.03 ^b^	2.04 ^b^	2.24 ^ab^	2.50 ^a^	2.40 ^ab^	2.09 ^b^	0.37
Viscosity	3.93 ^a^	3.61 ^ab^	3.46 ^b^	3.50 ^b^	3.43 ^b^	3.75 ^ab^	0.35
Body	3.80 ^a^	3.35 ^b^	3.41 ^ab^	3.43 ^ab^	3.18 ^b^	3.46 ^ab^	0.41
Cremosity	2.91 ^a^	2.66 ^abc^	2.51 ^bc^	2.45 ^bc^	2.38 ^c^	2.76 ^ab^	0.37

* Means with the same letter (^a–c^) on the same line do not differ at *p* ≤ 0.05 by Tukey’s test. ** Honestly significant differences (HSD) not MDS.

**Table 4:** Means * of parameters curves and standard deviation of the time–intensity analysis for sweetness (A) and bitterness (B).

**(A) Sweetness**						
**Parameter**	**Sucrose**	**Sucralose**	**Neotame**	**Stevia** **40% Reb. A**	**Stevia** **95% Reb. A**	**Blend**
I_MAX_	4.64 ^d^	5.25 ^c^	6.31 ^a^	5.85 ^b^	6.02 ^ab^	6.36 ^a^
T_IMAX_ (s)	15.15 ^a^	15.56 ^a^	15.53 ^a^	16.05 ^a^	15.54 ^a^	16.00 ^a^
T_TOTAL_(s)	24.76 ^b^	28.67 ^b^	33.26 ^a^	34.40 ^a^	34.77 ^a^	34.19 ^a^
Area	66.77 ^c^	92.88 ^b^	127.59 ^a^	118.03 ^a^	130.23 ^a^	129.28 ^a^
**(B) Bitterness**						
**Parameter**	**Sucrose**	**Sucralose**	**Neotame**	**Stevia** **40% Reb. A**	**Stevia** **95% Reb. A**	**Blend**
I_MAX_	1.58 ^e^	2.10 ^d^	2.61 ^c^	6.43 ^a^	5.87 ^b^	2.22 ^dc^
T_IMAX_ (s)	9.83 ^b^	11.62 ^b^	11.57 ^b^	14.25 ^a^	14.82 a	11.03 ^b^
T_TOTAL_(s)	14.73 ^b^	16.65 ^b^	19.40 ^b^	37.15 ^a^	36.51 ^a^	16.53 ^b^
Area	23.67 ^d^	30.74 ^cd^	41.20 ^c^	143.33 ^a^	126.02 ^b^	31.34 ^cd^

* Means with the same letter on the same line do not differ at *p* ≤ 0.05 by Tukey’s test. Reb. means rebaudioside.

**Table 5:** Means * of overall liking of consumer study.

Parameter	Sucrose	Sucralose	Neotame	Stevia 40% Reb. A	Stevia 95% Reb. A	Blend	HSD **
Appearance	5.89 ^a^	6.08 ^a^	6.20 ^a^	6.20 ^a^	6.05 ^a^	5.89 ^a^	0.57
Aroma	5.79 ^ab^	5.76 ^ab^	5.86 ^ab^	5.46 ^b^	5.44 ^b^	6.04 ^a^	0.58
Flavor	5.86 ^a^	5.36 ^a^	4.47 ^b^	2.58 ^c^	2.47 ^c^	5.54 ^a^	0.70
Texture	6.07 ^a^	5.94 ^a^	5.93 ^a^	5.05 ^b^	5.14 ^b^	6.08 ^a^	0.62
Overall Liking	5.83 ^a^	5.56 ^ab^	5.00 ^b^	3.24 ^c^	3.27 ^c^	5.66 ^a^	0.64

* Means with the same letter (^a–c^) on the same line do not differ at *p* ≤ 0.05 by Tukey’s test. ** Honest Significant Difference obtained in Tukey’s test.

## Data Availability

The authors declare that all the data supporting the findings of this study are available within the article.
